# Preparation and Characterization of Facilitated Transport Membranes Composed of Chitosan-Styrene and Chitosan-Acrylonitrile Copolymers Modified by Methylimidazolium Based Ionic Liquids for CO_2_ Separation from CH_4_ and N_2_

**DOI:** 10.3390/membranes6020031

**Published:** 2016-06-09

**Authors:** Ksenia V. Otvagina, Alla E. Mochalova, Tatyana S. Sazanova, Anton N. Petukhov, Alexandr A. Moskvichev, Andrey V. Vorotyntsev, Carlos A. M. Afonso, Ilya V. Vorotyntsev

**Affiliations:** 1 Nanotechnology and Biotechnology Department, Nizhny Novgorod State Technical University n.a. R.E. Alekseev, 24 Minina str., Nizhny Novgorod 603950, Russia; k.v.bogacheva@mail.ru (K.V.O.); mochalova_ae@mail.ru (A.E.M.); yarymova.tatyana@yandex.ru (T.S.S.); fox-off@mail.ru (A.N.P.); an.vorotyntsev@me.com (A.V.V.); 2 Department of Macromolecular Compounds and Colloid Chemistry, Lobachevsky State University of Nizhny Novgorod, 23 Gagarin av., Nizhny Novgorod 603950, Russia; 3 Institute for Problems in Mechanical Engineering, Russian Academy of Sciences, 85 Belinskogo str., Nizhny Novgorod 603024, Russia; triboman@mail.ru; 4 Research Institute for Medicines (iMed.UL), Faculty of Pharmacy, Universidade de Lisboa, Av. Prof. Gama Pinto, Lisboa 1649-003, Portugal; carlosafonso@ff.ulisboa.pt

**Keywords:** facilitated transport membranes, chitosan, modification, ionic liquid, copolymer, gas separation, atomic force microscopy (AFM)

## Abstract

CO_2_ separation was found to be facilitated by transport membranes based on novel chitosan (CS)–poly(styrene) (PS) and chitosan (CS)–poly(acrylonitrile) (PAN) copolymer matrices doped with methylimidazolium based ionic liquids: [bmim][BF_4_], [bmim][PF_6_], and [bmim][Tf_2_N] (IL). CS plays the role of biodegradable film former and selectivity promoter. Copolymers were prepared implementing the latest achievements in radical copolymerization with chosen monomers, which enabled the achievement of outstanding mechanical strength values for the CS-based membranes (75–104 MPa for CS-PAN and 69–75 MPa for CS-PS). Ionic liquid (IL) doping affected the surface and mechanical properties of the membranes as well as the gas separation properties. The highest CO_2_ permeability 400 Barrers belongs to CS-b-PS/[bmim][BF_4_]. The highest selectivity α (CO_2_/N_2_) = 15.5 was achieved for CS-b-PAN/[bmim][BF_4_]. The operational temperature of the membranes is under 220 °C.

## 1. Introduction

Chitosan (CS), poly[β(1→4)-2-amino-2-deoxy-d-glucopyranose], is a linear amino polysaccharide obtained by the deacetylation of chitin, an abundant natural polymer found in fungal cell membranes and in the exoskeleton of invertebrate species (crustaceans). Some of the most attractive features of these materials are their biodegradability, biocompatibility, flocculation [1], and non-toxicity while forming clear films with impressive thermal properties [1,2,3,4,5,6,7]. Natural selection over the course of millions of years has created a membrane as the perfect tool for the separation of substances in living organisms, however, direct use of biological membranes in the laboratory and industrial processes is currently impossible, mostly due to their low mechanical strength [8,9]. In spite of the fact that CS is a promising matrix for carbon dioxide and other acidic gas separation from flue gases through the amino group in its repeating unit, it has not found widespread use in gas separation thus far. One of the main challenges is the formation of materials with low physical-mechanical properties and low stability due to the hydrophilic character of the surface and the pH sensitivity of CS, which is ill-suited as a component for gas separation modules [2,9]. Moreover, despite the selectivity for CO_2_ separation achieved by CS, it has low permeation caused by the high crystallinity of this polymer [10]. Thus, judicious selection of efficient and appropriate modifications is needed to fully explore potential gas separation capabilities.

CS properties can be further improved mainly using two approaches: direct CS chemical and/or physical modification, and formation of composite membranes; however, in some cases, both methods are used simultaneously [7,10,11,12,13,14,15]. A notable change in CS gas separation properties was achieved by moistening the original CS membranes [11,12,13]. Grulke and El-Azzami have shown that the permeation and separation of gases is enhanced significantly for water-swollen CS membranes due to reaction mechanism between CO_2_, amino groups, and water plasticizing properties [11]. The maximum CO_2_ permeability was 482 Barrers at a selectivity of α(CO_2_/H_2_) = 43, whereas a selectivity of α(CO_2_/N_2_) = 250 was obtained for swollen membranes at 110 °C and 1.5 atm feed pressure. Ito *et al.* demonstrated a new approach by separation of CO_2_/N_2_ in humidified feed gas [11]. The CO_2_ permeability was up to 100 Barrers with selectivity up to 100 at a feed pressure of 3.5 atm. Bae *et al.* discovered that maintaining water in chitosan membranes gives higher CO_2_ permeability and selectivity, even at higher temperatures [13]. The permeation of pure CO_2_ and N_2_ through wet CS membranes at 20 and 30 °C for a feed pressure up to 20 atm showed CO_2_ permeability under 1 Barrer with selectivity up to 37. In comparison to water-swollen CS membranes, Grulke and El-Azzami also modified CS by blending with solutions containing arginine salt solutions for obtaining gas separation membranes [14]. Arginine salt–CS membranes achieved high CO_2_ transport properties at operating temperatures and pressures suitable for industrial applications such as fuel cell processes and flue gas purification, and exhibited carbon dioxide transport properties about three times more favorable than those for swollen CS membranes.

Crosslinking presents another well-established method of CS modification for gas separation application [15]. Crosslinked CS membranes were prepared by Shude Xiao and co-workers from interfacial crosslinking of CS membranes in trimesoyl chloride (TMC)/hexane [16]. The membrane developed showed a CO_2_ permeability of ~163 Barrers and an optimized selectivity for CO_2_/N_2_ of approximately 42. Beyond this technology, composite gas separation membranes containing CS layers with good separation properties have been developed by different research groups; Kouketsu *et al*. prepared a poly(amidoamine) dendrimer composite membrane containing a CS gutter layer [17]. The membrane showed very promising results with a selectivity as high as 400 for CO_2_/N_2_ mixtures, and a CO_2_ permeance of 0.12 × 10^−5^ GPU. Shen and co-workers obtained the carboxymethyl CS/polyethyleneimine sandwich membranes with polysulfone ultrafiltration membranes as a support layer for the separation of CO_2_/N_2_ mixtures [18]. The dry membranes showed a high permeance of CO_2_ of 2.1 GPU and the ideal selectivity for the CO_2_/N_2_ mixture of about 33. Shen *et al.* fabricated a facilitated transport mixed matrix membrane based on polyvinyl amine and CS as the polymer matrix, and coated this material on to a porous polysulfone support, graphene oxide (GO), grafted with hyperbranched polyethylenimine (HPEI-GO) which was added as a nanofiller [19]. It was shown that CO_2_ transport through the membrane occurs mainly by an assisted facilitated transport mechanism by a solution-diffusion mechanism. The highest CO_2_ permeance (36 GPU) was achieved with 2 wt % HPEI-GO membranes, and CO_2_/N_2_ selectivity reached 107 in 3 wt % HPEI-GO membranes. Furthermore, Assis and Hotchkiss studied CO_2_/O_2_ permeability through the CS film modified by a thin hydrophobic silicon coating [20] which resulted in 0.4 Barrer CO_2_ permeation through the membrane. Bai *et al.* obtained a CS-acetic acid and CS-polymer complex gas separation membrane, in which the CS-acetic acid complex membrane showed high permselectivities for oxygen and carbon dioxide [21]. Complex membranes with synthetic polymers such as polyvinyl alcohol, polyacrylamide, and polyvinylpyrrolidone were also studied for CO_2_ separation. In comparison with that of CS-acetic acid complex membrane permeation, CS-polymer complex membranes permeation increased, while selectivity slightly decreased.

Through an intensive search for new methods of CO_2_ sorption, a novel class of materials referred to as ionic liquids (IL) was discovered to display efficient CO_2_ selective capture [22,23,24,25,26]. ILs are cation/anion pairs with a melting point below 100 °C [22], and their unique properties make them promising modifiers for gas separation membranes that enable CO_2_ separation levels not possible with pristine or conventional materials. Owing to these properties, ILs have attracted much attention in recent gas separation research [7,27,28,29,30,31]. Albo and coworkers made a great contribution to the gas separation properties study of the systems containing IL [27,28,29]. In these works it was shown that the IL might be used as the solvent in the cross-flow and parallel membrane contactors for CO_2_ as well as SO_2_ separation providing a high efficiency of the process. CS-based membranes were also modified by introduction of ILs. Casado-Coterillo prepared and tested novel mixed matrix membranes composed of IL/CS, ETS-10/CS, and ETS-10/IL/CS for CO_2_ and N_2_ permeation applications [7]. Three-component membranes showed the highest solubility selectivity for CO_2_/N_2_, displaying values of 38.48.

CS has already been tested in pervaporation for dehydration of solvents, such as ethanol, isopropanol, tetrahydrofuran, and acetone, with high separation performance in terms of selectivity and water flux, as well as for gas separation [32,33,34]. Similar approaches for CS modification are used in pervaporation membrane development [35,36,37,38,39]. Likewise, attention has been given to materials based on CS copolymers with synthetic monomers [40,41], while they have not found a proper study for gas separation. At the same time, copolymerization with vinyl monomers is one of the most promising approaches to modify CS thanks to their facile implementation which may be applied to a wide range of monomers, with notable influence on mechanical properties, solubility, thermal properties, and other important characteristics [42,43,44]. In the past decade, it has been seen that CS copolymerization with acrylonitrile (AN) [45,46,47,48] and styrene (S) [49,50,51] provides significant advantages in the obtained physiochemical properties. Moreover AN and S are large-capacity monomers and there polymerized properties are well studied. Copolymerization with AN and S would provide a stable polymer matrix resistant to mechanical and thermal influence. However, it is likely, the level of the free volume in such a matrix would not be high as a result of high crystallinity, and as a consequence, low permeation ability is expected. In order to increase the free volume and adsorption capacity of CO_2_ it is proposed to dope original copolymer with ILs. We used a commercially available IL based on the cation 1-butyl-3-methylimidazolium ([bmim]) with three different anions: tetrafluoroborate ([BF_4_]), hexafluorophosphate ([PF_6_]) and bis((trifluoromethyl)sulfonyl) imide ([Tf_2_N]). It is known that the nature of the cation does not significantly affect the sorption properties of the ionic liquid, while the anion plays a major role [52]. Therefore the cation was chosen arbitrarily. The literature shows that ILs containing the acetate anion possess a high degree of absorption for CO_2_ across a wide range of temperatures and applied pressures [53,54]. Due to the preparation technique of the membranes in this work, acetate ions already exist in the polymer matrix. A range of anions including [BF_4_], [PF_6_], [Tf_2_N] with medium sorption capacity and various anion radius and intensities of molecular interaction with water were chosen with the aim of comparison with the literature data.

The scope of the present work is focused on the preparation and gas separation study of facilitated transport membranes based on CS graft and block copolymers with poly(acrylonitrile) (PAN) and poly(styrene) (PS) doped with IL. IL doping into CS matrix leads to the decrease in crystallinity of the biopolymer, and consequently results in an increase in permeation without the loss of selectivity [7,55,56,57].

## 2. Results and Discussion

### 2.1. Structural Properties

**Figure 1:**
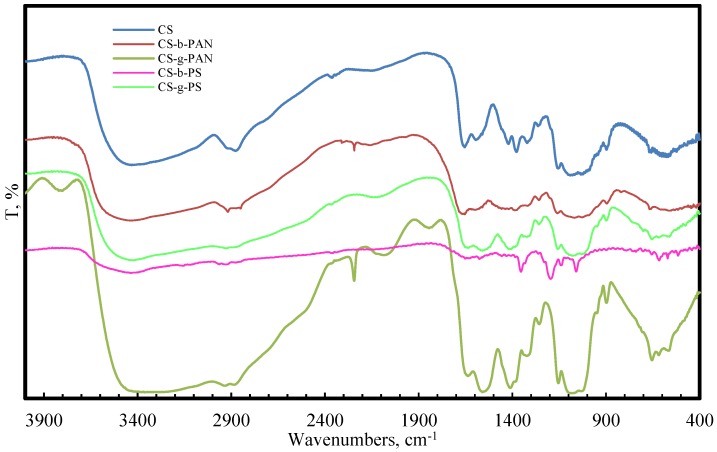
FTIR spectra of pure CS, CS-PAN copolymers, and CS-PS copolymers.

Fourier transform infrared spectroscopy analysis (FTIR) of the copolymer samples in comparison to pure CS were used to support the successful modification of CS. FTIR spectra of the copolymers, purified by homopolymer extraction, are presented in Figure 1.

The FTIR spectra of chitosan/poly(acrylonitrile) (CS-PAN) copolymers arise from the frequencies of the C≡N functional group (2241 cm^−1^) [58], while the chitosan/poly(styrene) (CS-PS) copolymers FTIR spectra shows bands corresponding to the stretching vibrations of the benzene ring (1570 cm^−1^) [59]; these findings support CS-PAN and CS-PS copolymer formation. The CS main bands were also detected in copolymer spectra, and the absorption bands at 1151–1180 cm^−1^ were assigned to the anti-symmetric stretching of a C–O–C bridge. A peak at 2918–2925 cm^−1^ corresponds to a symmetric –CH_2_ stretching vibration, and the signal at 1377–1384 cm^−1^ was assigned to a methyl stretch of the amine group. Peaks at 1633–1652 cm^−1^ and 1540–1590 cm^−1^ correspond to C=O stretches (amide I) and NH stretching (amide II), respectively [60,61].

**Figure 2:**
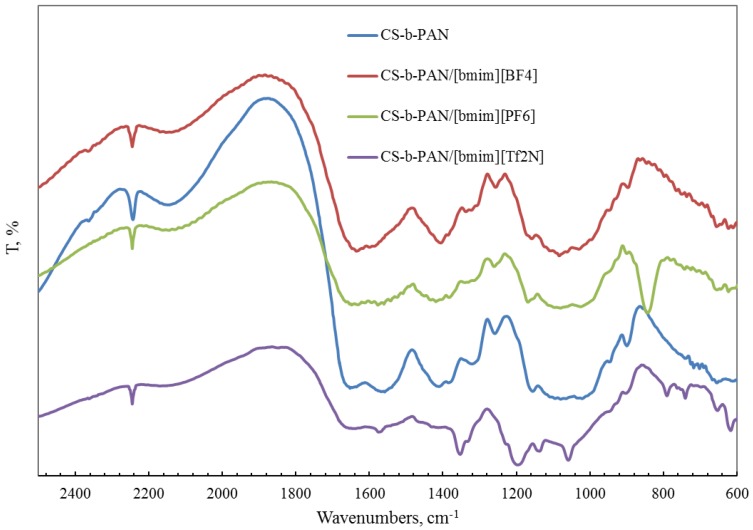
FTIR spectra of pure CS-b-PAN copolymer and CS-b-PAN copolymers doped with IL.

**Figure 3:**
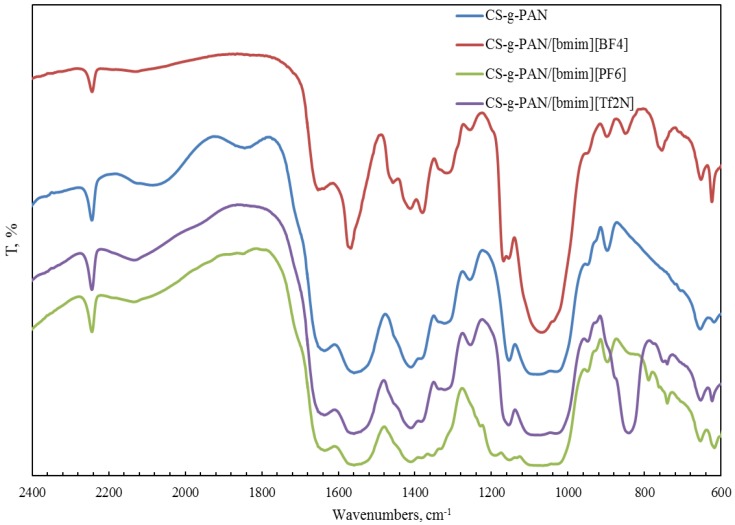
FTIR spectra of pure CS-g-PAN copolymer and CS-g-PAN copolymers doped with IL.

**Figure 4:**
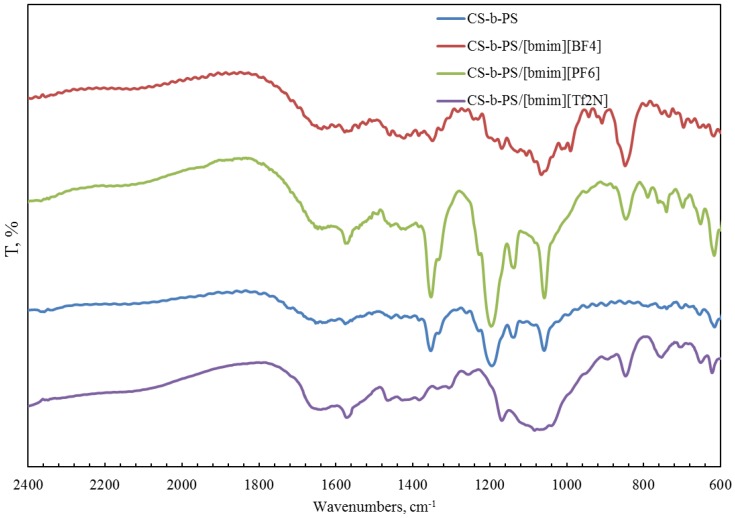
FTIR spectra of pure CS-b-PS copolymer and CS-b-PS copolymers doped with IL.

**Figure 5:**
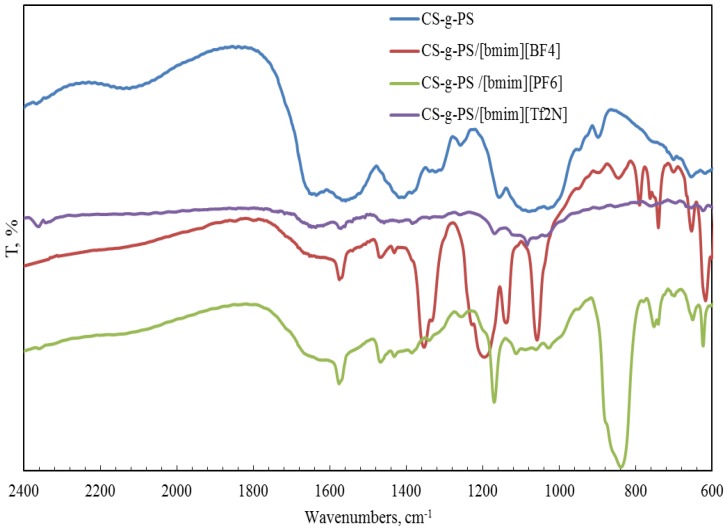
FTIR spectra of pure CS-g-PS copolymer and CS-g-PS copolymers doped with IL.

FTIR spectroscopy was also used for the characterization of a copolymer–ionic liquid interaction. The obtained spectra of chitosan block copolymer with poly(acrylonitrile) (CS-b-PAN) doped with ionic liquids (IL), chitosan graft copolymer with poly(acrylonitrile) (CS-g-PAN) doped with IL, chitosan block copolymer with poly(styrene) (CS-b-PS) doped with IL, and chitosan graft copolymer with poly(styrene) (CS-g-PS) doped with IL are shown in Figure 2, Figure 3, Figure 4 and Figure 5.

In the CH stretching region, the characteristic aliphatic symmetric CH stretching band is observed at 2878 cm^−1^, whereas the asymmetric aliphatic bands appear at 2939 and 2970 cm^−1^. The CH stretching bands of the [bmim] ring appear at 3132 cm^−1^ with a shoulder around 3105 cm^−1^ and at 3160 cm^−1^. For CS-PAN, incorporation of all three ILs resulted in shifts of the peaks *v*_ass_(C–O–C) on the order of 2–7 cm^−1^, and peak shifts of *v* (C≡N) from 2–5 cm^−1^. In all cases, three types of ionic liquids based on cations [bmim]^−^ are built into the structure of our samples.

### 2.2. Surface Properties

**Figure 6:**
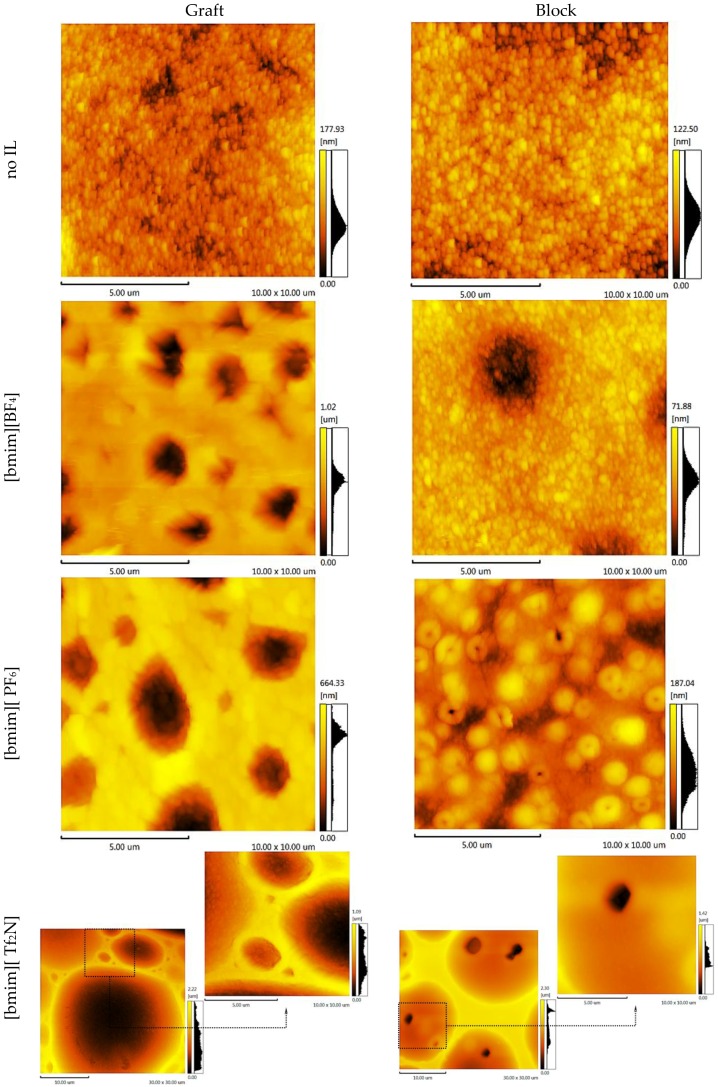
AFM images of the CS-PAN copolymers.

**Figure 7:**
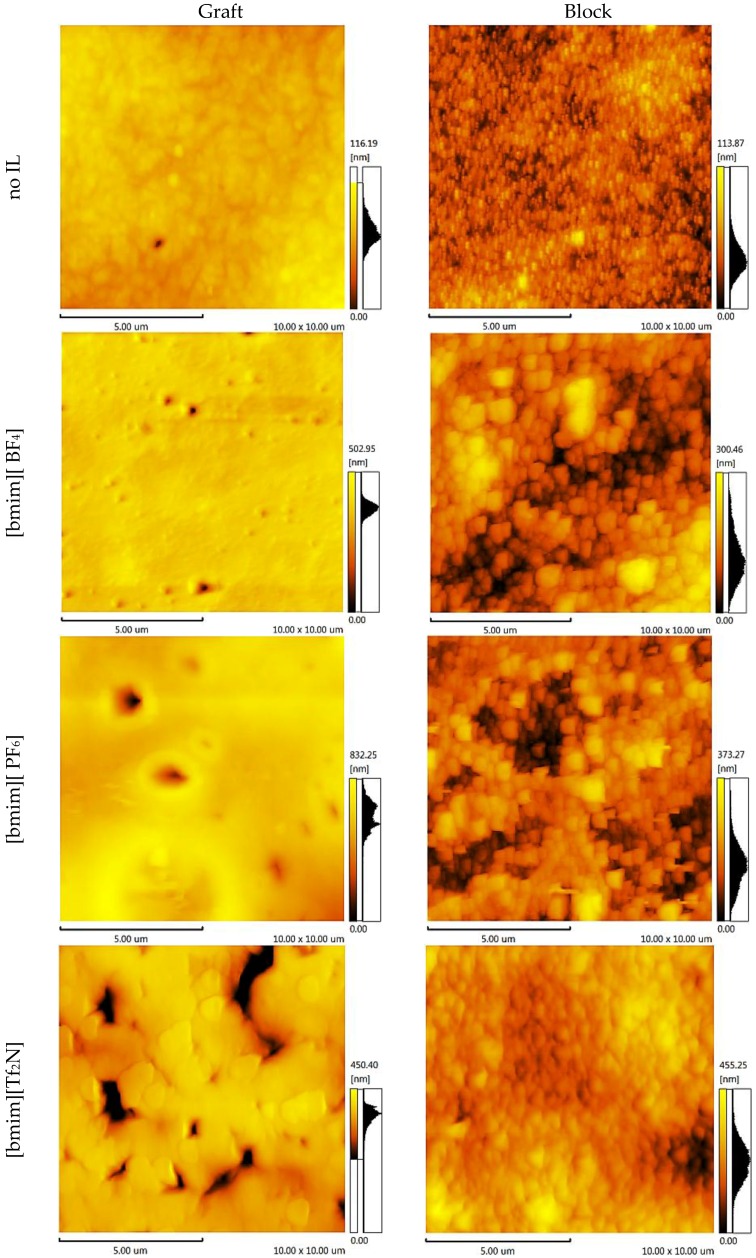
AFM images of the CS-PS copolymers.

Knowing the membrane surface topography and the structural units’ statistical distribution enables the simulation of the gas separation process through the polymer membrane. To elucidate these properties, atomic force microscopy topology analysis (AFM) was conducted. The AFM images of the CS-PAN and CS-PS copolymers are shown in Figure 6 and Figure 7, respectively. Two important components of the surface roughness parameters were determined: mean roughness (*R_a_*, the mean value of the surface relative to the center plane), and the mean difference between the highest peaks and lowest valleys (*R_z_*). The results are shown in Table 1. 

From the results of the microscopy analysis, it can be concluded that the pure copolymers possess nodular microstructures, wherein nodules are observed as bright high peaks. The CS-g-PS has the most densely-packed surface structure. The detailed analysis of the membrane surface AFM profiles leads to the conclusion that there are no pores, thus membranes are non-porous.

Despite IL doping yielding a different influence on CS-PAN and CS-PS copolymer surfaces, generally, the incorporation of the ionic liquids in the copolymer matrix causes the formation of an explicit macro-relief, thus, the nodular structure remains in those samples at the micro level. The specific impact of [bmim][BF_4_] and [bmim][PF_6_] on the surface structure is more pronounced for CS-PAN then for CS-PS. This is likely related to the fact that PAN polymer chain mobility is higher than PS. For the same reason, block copolymers are more resistant to the effects of IL doping in comparison to graft copolymers. The IL [bmim][Tf_2_N] has the greatest influence on the copolymer structure due to the more sterically demanding anion. The nodule surface structure may be related to a particular orientation of macrochain fragments when forming a film. Similar macro-chains twist, forming areas of various chemical natures. The effects of the monomer units’ polarity and the IL hydrophilic/hydrophobic properties on IL distribution between macromolecular chains are far from trivial and should not be ignored.

The observed microstructure is formed due to the specific orientation of macromolecular chains in film formation. To determine the packing trend of copolymers, the surface chemical nature was investigated by wettability measurements. It is known that CS is hydrophilic and has good wettability of polar solvents, at the same time opposite properties are peculiar for the synthetic macrochain fragments. Since CS swells in water, CH_2_I_2_, which does not interact with copolymer matrices, was selected as test liquid to obtain adequate data.

The sample’s surface has good wettability with CH_2_I_2_, a relatively nonpolar liquid: cosθ = 0.807 in the case of CS-b-PAN and cosθ = 0.724 in the case of CS-g-PAN; cosθ = 0.699 in CS-b-PS and; cosθ = 0.755 for CS-g-PS. Since the surface has good wettability with nonpolar liquid, it can be concluded that the observed micro-relief is formed by orienting the synthetic polymer fragments to the surface and within the scope of the CS film.

Furthermore, to determine the compatibility of copolymers with ILs the wettability of membranes with ILs was studied. It was found that all copolymers liquophilic for ILs, and wettability increases in the following order: [bmim][BF_4_] < [bmim][PF_6_] < [bmim][TF_2_N] in all cases.

### 2.3. Thermal Properties

**Figure 8:**
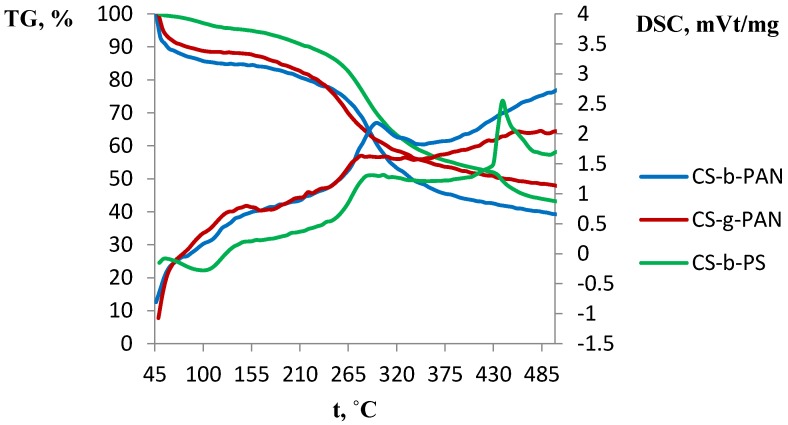
DSC and TGA curves of CS-g-PAN, CS-b-PAN, and CS-b-PS copolymers.

**Figure 9:**
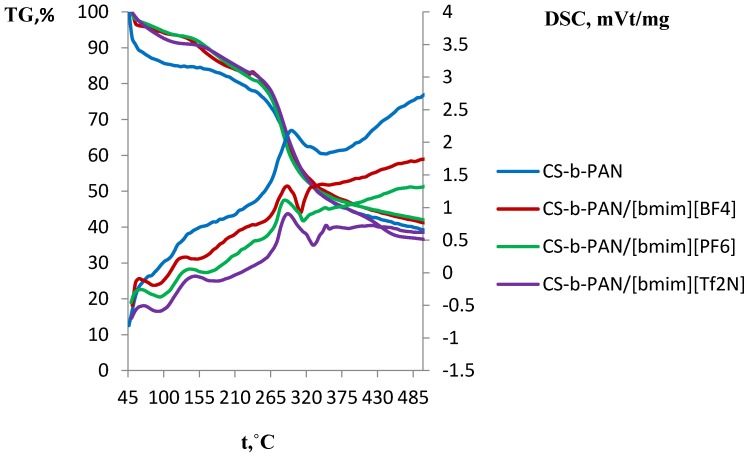
DSC and TGA curves of CS-b-PAN, CS-b-PAN/[bmim][BF_4_], CS-b-PAN/[bmim][PF_6_], and CS-b-PAN/[bmim][Tf_2_N] copolymers.

A number of industrial gas separation processes are carried out at elevated temperatures, thus it is necessary to study the membrane material’s thermal properties. The thermal properties of the obtained films are shown in the gravimetric analysis (TGA) and differential scanning calorimetry (DSC) diagrams presented in Figure 8 and Figure 9, respectively. To describe thermal properties of the obtained materials, original copolymers and those doped with IL were studied. The major weight loss for CS-PAN (Figure 8) occurs at a temperature between 220 and 380 °C. This weight loss is due to the decomposition of the polymer matrix as supported by the presence of a sharp peak in the DSC curve.

This effect is more explicit for the graft copolymer than for the block copolymer, due to the higher stability of the block copolymer matrix as a result of its structure. Similar effects were observed for the surface properties of copolymers, as well as for the mechanical properties. It should be noted that copolymers typically have lower decomposition temperature than pure CS (280 °C) [7]. Here, it is seen that a significant impact of PAN incorporation occurs in which decomposition according to the literature starts at 220–230 °C [62]. Conversely, a different behavior is observed for the copolymer with PS (Figure 8). The TGA curve for this copolymer has two regions of weight loss: 220–420 °C and 420–490 °C. This likely indicates PS decomposition (pure PS decomposes at 260 °C) [62] followed by CS decomposition. The DSC curve shape of the copolymers doped with IL differs significantly from the corresponding curve for the original copolymer (Figure 9). Additional mass-spectrometry analysis following the thermal gravimetric analysis and differential scanning calorimetry showed that the ionic liquids boil noticeably above the decomposition temperature. Decomposition of fluorinated ILs occur in the range of 311–350 °C, wherein HF, CH_3_F, and C_4_H_9_F were observed as decomposition products.

### 2.4. Mechanical Properties

The influence of IL doping on the mechanical properties of the copolymers was investigated by tension elongation measurement. The results are presented in Table 2.

Better mechanical properties of CS-PAN copolymers in comparison to CS-PS copolymers are expected as PAN has greater tensile strength than PS [63]. It was also predicted that ILs will serve as a plasticizer for CS due to mutual solubility [53], however, a lack of elongation growth with the decrease in tensile strength indicates the opposite relationship. It should be noted that the IL with [Tf_2_N] anion yields an adverse effect on the CS-PAN mechanical properties, while for the CS-PS, augmentation is observed. In general, physiomechanical properties of block copolymers are more resistant to changes, which correlate to the aforementioned hypotheses.

### 2.5. Gas Separation Properties

For the investigation of gas separation properties, gas combinations such as carbon dioxide and methane were chosen because of their potential application for removal of acidic gases at the essential stage of natural gas and biogas treatment. In addition, the separation of carbon dioxide and nitrogen was investigated owing to the importance of this process to the capture of carbon dioxide from power plant flue gases [64]. Among the traditional methods commercially available for acidic gas separation, the most common methods include absorption by physical and chemical adsorbents (e.g., amine solutions, carbonates), cryogenic distillation, membrane separation, and catalytic oxidation [65]. In general, all these methods have a specific field of application limited by the sorption capacity, regeneration, and stability to high pressures. The novel trend in gas stream treatment, however, is judicious selection of enhanced solvents for aggressive gases based on sorption of CO_2_ using IL-based hybrid materials [26,30,31,66,67,68,69,70].

In the present work, relatively high mechanical strain, thermal stability, and chemical stability achieved by copolymerization are presented. These properties naturally lend themselves to testing of these materials as gas separation membranes. Carbon dioxide has two polar bonds C=O, nitrogen as a simple non-polar gas, and methane as a hydrocarbon, non-polar gas, were chosen for permeability measurements. Pure nitrogen was used as a test gas to detect changes in membrane structure before and after carbon dioxide permeability.

**Figure 10:**
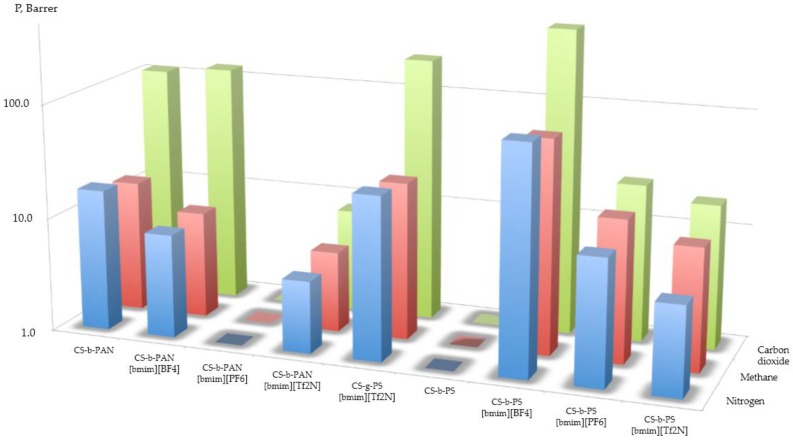
The permeability coefficients.

The aforementioned examples of separation by materials doped with IL were promising for carbon dioxide separation; unfortunately, some of the synthesized copolymers were unable to be used in permeability tests. With the exception of CS-g-PST doped by [bmim][Tf_2_N], all graft copolymers were destroyed under increased pressure. The values of the obtained permeability coefficients for these experiments are presented in Figure 10, and the obtained results are compared with data discussed in the introduction [11,12,13,14,15,16,17,18]. The selectivity values presented in Table 3 are roughly the same for carbon dioxide/methane and carbon dioxide/nitrogen systems. As shown here, only block-copolymers which were doped with IL were permeable for carbon dioxide, methane, and nitrogen gases lower permeability in the current experiments. The most permeable film was made of CS-b-PS with [bmim][PF_6_]. This observation leads us to conclude that the permeability data has a correlation with data obtained from AFM measurements presented in Figure 6 and Figure 7. For CS-b-PAN doped by IL, the permeability and selectivity were increased in the following order [bmim][Tf_2_N] < [no IL] < [bmim][BF_4_]. For CS-b-PS, the permeability and selectivity increases in the order of the following IL: [bmim][Tf_2_N] < [bmim][PF_6_] < [bmim][BF_4_]. This is evidence that the value of solubility of carbon dioxide is affected *vice versa* to the permeability of mass-transfer mechanism.

We suggest that the CO_2_ facilitated transport mechanism in obtained membranes occurs in two steps, but mostly through the IL ions, and macromolecular fragments with protonated amino group and acetate anion, due to the presence of acetic acid in the reaction medium, that appears to be a result of Coulombic interaction. The CO_2_ sorption by the IL is suggested to be the first step of the proposed facilitated transport mechanism. Obtained membranes were found to be non-porous membranes by means of AFM, as mentioned above. Thus, it is necessary to provide a target gas sorption by the membrane material for separation of it from the smaller molecules, such as N_2_. In our case, the presence of IL in the matrix of the membrane copes with this problem. Both the nature of the cation and anion impact the dissolution of CO_2_, however the anion nature is known to be the dominating factor because of the Lewis acid–base type interaction where the anion acts as a Lewis base, and CO_2_ as a Lewis acid. Brennecke *et al.* found that the solubilities of CO_2_ in the ILs are in the order of the anions: [BF_4_]^−^ ≈ [PF_6_]^−^ < [Tf_2_N]^−^ [71]. It should be noted that the system of ions with adsorbed carbon dioxide molecules is not absolutely rigid, but rather flexible due to the mobility of macromolecular chains. Berne *et al.* proposed that molecular dynamics simulation shows the introduction of CO_2_ occurring in the positions above and below the imidazolium rings, or close to the long alkyl chains on the rings [72]. The diffusion coefficient calculations performed by Berne *et al.* showed that CO_2_ mobility is higher than [bmim]^+^ and [PF_6_]^−^, thus, CO_2_ molecules diffuse through the fluid network formed by IL ions, acetate anion, and protonated amino groups of CS—the facilitated transport mechanism in the gas separation membrane is thereby proposed to occur by such a process. Thus, further sorbed gas travels through the ion grid formed by the protonated amino group, acetate anion and IL ions, that appears to be a result of Coulombic interaction. Facilitated transport of carbon dioxide is also promoted by the macromolecular chain mobility due to the pressure difference in the experimental unit. The described mechanism may be also called facilitated transport with fixed site carrier, and similar cases have been described in the literature [11].

The structure of this membrane shows micro-reliefs which better facilitate gas separation. In the case of copolymers with [bmim][Tf_2_N], higher density is apparent which also correlates with the observed permeability data. The fact that copolymers without any IL are found to be impermeable leads us to conclude that facilitated transport through the membrane is realized with the assistance of the IL inside the membrane, and the state of it is influenced by the transmembrane transport. The relatively small values of selectivity (ratio of permeability coefficients) suggest the presence of a non-solution-diffusion mechanism. For the carbon dioxide, reactively methane is a fast impurity, but nitrogen is a slow impurity for methane. Usually, membrane gas separation separates gas flow for fast impurities [73], but also there are some techniques, which allow separation of faster impurities from the target gas flow [74,75]. Herein, we can conclude that the obtained membranes, despite the low separation ability, have potential utility for special applications (gases high separation for semiconductor need *etc.*), moreover, a facile approach to the synthesis of a wide range of CS copolymers has been presented.

## 3. Materials and Methods

### 3.1. Materials

CS (“Bioprogress” corp., Shchyolkovo, Moscow region, Russia) with molecular weight 1.05 × 10^5^ and deacetylation degree 80% was used without further purification. Acrylonitrile monomer was obtained from ”Saratovorgsintez” (LUKIOL PJSC Subsidiary, Saratov, Russia) and was purified by drying with NaOH, followed by distillation. Styrene (99%, Sigma-Aldrich, St. Louis, MO, USA) was used after distillation. Solvents of acetone, tetrahydrofuran, dimethylformamide, and acetic acid (Sigma-Aldrich, St. Louis, MO, USA) were used after distillation.

### 3.2. Copolymer Synthesis

Graft copolymers CS-g-PAN and CS-g-PS were obtained in 1.5% water-acetic acid polysaccharide solutions with 1.2% acetic acid content. The quantity of monomer in the reaction medium was 0.62 mol/L. The copolymerization technique was developed and characterized by Baranov, *et al.* [58]. Block copolymer synthesis for CS-b-PAN and CS-b-PS was performed according to published procedures [59,76]. The reaction medium consisted of 3% water-acetic acid polysaccharide solutions with 6% acetic acid content, ascorbic acid, and hydrogen peroxide in the ratio [C_6_H_8_O_6_]:[H_2_O_2_] = 1:1, [CS link]:[H_2_O_2_] = 100 (for acrylonitrile) and 50 (for styrene), and synthetic monomer content concentration of acrylonitrile was 0.152 mol/L, styrene, 0.087 mol/L.

### 3.3. Fourier-Transform Infrared Spectroscopy Analysis

Copolymers were obtained after Soxhlet extraction for 48 h, and characterization performed by FTIR spectroscopy of the corresponding samples on a FTIR spectrometer (IRrafinity-1 (Shimadzu, Kioto, Japan)) at ambient temperature. A minimum of 30 scans was signal-averaged with a resolution of 4 cm^−1^ at the 4000–400 cm^−1^ range. All other parameters were not controlled and corresponded to the testing characteristics established by the manufacturer. The sample measurements were carried out in film samples treated in a potassium bromide matrix. Samples for FTIR were prepared by cooperative pressing of copolymers and dehydrated KBr powder at pressure 100 MPa under vacuum at 25 °C.

### 3.4. IL Doping

IL: [bmim][BF_4_], bmim][PF_6_], and [bmim][Tf_2_N] were purchased (Sigma-Aldrich) and used without further purification for doping CS matrices. For the IL doping, 25 mL of the reaction media after copolymer synthesis was placed into a two-neck round-bottom flask equipped with stirrer. The estimated amount of IL (10% from polymer mass in reaction media) was added into the flask dropwise under argon atmosphere. The process was carried out for two hours under stirring and ambient temperature.

### 3.5. Membrane Preparation

Membranes were prepared by casting solutions onto poly(ethylene terephthalate) films in a laboratory dryer. The initial thickness of the cast film was adjusted using an applicator-casting knife. Evaporation was conducted at room temperature and 1 atmosphere for 2–3 days, followed by desiccation under vacuum for 3 h at room temperature. After desiccation, the materials were characterized and used for gas permeation experiments.

### 3.6. Atomic Force Microscopy Topology Analysis

The topography of membrane surfaces was determined by atomic force microscopy (AFM). Atomic force microscope Shimadzu SPM-9700 (Japan) with scanner 30 μm was used in the force modulation mode. As a tip, commercially available silicon tip POINTPROBE FMR-20 S/N-71814F8L882 (Nano World Innovative Technologies, Matterhorn, Switzerland) was used with spring stiffness 1.3 N/m and the radius of the curve of the tip typically not more than 8 nm and guaranteed to be not more than 12 nm. The length of the tip was 15 μm. Scan sizes were 10 × 10 μm^2^ and 30 × 30 μm^2^. The surface characterization was carried out at ambient temperature. The samples were cleaned of dust with ethanol before measurement, and then affixed to the center of the sample holder using two-sided carbon tape (SPI Supplies Division of STRUCTURE PROBE Inc., West Chester, PA, USA). After image acquisition, the arithmetic average roughness height (*R_a_*), was obtained by a program in the AFM image processing toolbox (SPM Online, Version 4.02, Shimadzu, Kyoto, Japan). The accuracies of obtained values were equal to 0.01 nm for average roughness *R_a_*.

The cantilever with a curvature radius of tip not more than 8 nm was used for the observation of a topographic map to minimize the error introduced by the cantilever due to the narrowing of profile recesses.

Two important components of the surface roughness parameters were determined, including mean roughness (*R_a_*, the mean value of the surface relative to the center plane) and the mean difference between the highest peaks and lowest valleys (*R_z_*) using software SPM Manager ver. 4.02 (Shimadzu, Japan).

### 3.7. Study of the Surface Chemical Nature

The samples’ surface chemical nature was investigated by membrane wettability with test liquid (diiodomethane) using the contact angle (cosθ) measurement method. A drop of the test liquid was applied on the sample surface. The drop’s diameter (*d*) and height (*h*) determination was conducted after the drop reached its equilibrium state. The cosine of the contact angle was calculated according to the formula:

(1) $$\cos\theta =\frac{{\left(\frac{d}{2}\right)}^{2}-h^{2}}{{\left(\frac{d}{2}\right)}^{2}+h^{2}}$$

### 3.8. Tension Elongation Measurement

The membranes’ mechanical properties—breaking strength (σ), MPa, and relative elongation (ε), %—were determined on a Z005 tensile machine (ZWICK, Ennepetal, Germany) at a tension rate of 50 mm/min.

### 3.9. Thermogravimetric Analysis

For thermal gravimetric analysis (TGA) and differential scanning calorimetry (DSC) measurements, samples between 5 and 8 mg were placed in open alumina pans and experiments were conducted using an STA 449 F1 Jupiter (NETSCH, Selb, Germany) thermal analyzer. The thermograms were recorded in the range of 50–1500 °C and with a ramping rate of 10 °C/min in an atmosphere of argon.

### 3.10. Gas Permeation Tests

The single gas permeability coefficients of nitrogen, carbon dioxide, and methane through the obtained membranes were measured by the apparatus proposed by Crespo *et al.* [30,77,78] at 25–26 °C. Permeability coefficients (P) (1 Barrer = 3.348·10^−16^ mol·m·m^−2^·s^−1^·Pa^−1^) were calculated according to the method proposed in [30,77,78], but were improved by the experimental set-up by automatization of the permeability measurement process, and by further calculations through the use of programming logic controller (Unitronix, Airport City, Israel). The errors of permeability measurements were less than 10%. Ideal selectivity (α) for pairs of gases was calculated as a ratio of its permeability coefficients.

## 4. Conclusions

Novel gas separation facilitated transport membranes were prepared by doping methylimidazolium based ionic liquids into original polymer matrices synthesized by graft and block copolymerization of CS with styrene and acrylonitrile. IL doping affected the surface and mechanical properties of the membranes as well as the gas separation properties. By means of atomic force microscopy, it was found that the obtained membranes were non-porous with well-developed surface organized at the macro and micro level. Despite the fact that the original copolymers show high values of mechanical strength, not all membranes were suitable for gas separation testes due to a sharp decline in mechanical properties with the addition of IL; however, we took the risk for the sake of gain in selectivity and permeability. The CO_2_ facilitated transport with the fix carrier mechanism was proposed based on the results of the gas permeability study, the copolymer structure, surface and mechanical properties, as well as literature analysis. Investigation of the thermal properties of the obtained membranes shows a permissible operating temperature up to 220 °C before polymer decomposition occurs. The membranes prepared exhibit potential utility in specialized applications such as high purification of gases, despite the low separation capacity. The synthetic protocols presented within this manuscript additionally provide a versatile approach to the efficient preparation of CS copolymers bearing diverse functionality.

## Figures and Tables

**Table 1:** The surface roughness parameters.

IL	CS-PAN				CS-PS			
	Graft		Block		Graft		Block	
	*R_a_*, nm	*R_z_*, nm	*R_a_*, nm	*R_z_*, nm	*R_a_*, nm	*R_z_*, nm	*R_a_*, nm	*R_z_*, nm
no	16.03	88.06	13.11	60.14	8.22	45.57	9.81	53.29
[bmim] [BF_4_]	106.07	470.57	7.80	35.12	19.80	230.69	41.36	149.08
[bmim] [PF_6_]	112.28	325.72	23.29	86.97	72.03	393.60	42.34	168.30
[bmim] [Tf_2_N]	228.53	546.18	141.58	707.65	55.33	435.08	50.27	233.43

**Table 2:** The mechanical properties of the obtained materials.

IL	CS-PAN				CS-PS			
	Graft		Block		Graft		Block	
	σ, MPa	ε, %	σ, MPa	ε, %	σ, MPa	ε, %	σ, MPa	ε, %
no	75.57	1.49	104.29	2.80	68.84	0.43	75.15	2.06
[bmim] [BF_4_]	12.71	0.74	78.91	2.30	8.36	0.31	42.54	2.03
[bmim] [PF_6_]	28.29	1.41	80.19	2.37	6.62	0.39	53.19	2.36
[bmim] [Tf_2_N]	17.35	0.79	68.97	2.39	18.96	1.06	59.72	2.36

**Table 3:** The selectivity of the obtained materials.

IL	CS-b-PAN		CS-g-PS		CS-b-PS	
	CO_2_/CH_4_	CO_2_/N_2_	CO_2_/CH_4_	CO_2_/N_2_	CO_2_/CH_4_	CO_2_/N_2_
no	7.8	6.3	n/c	n/c	n/c	n/c
[bmim] [BF_4_]	14.6	15.5	n/c	n/c	6.2	4.8
[bmim] [PF_6_]	n/c *	n/c	n/c	n/c	1.3	1.9
[bmim] [Tf_2_N]	1.6	1.9	8.6	7.6	1.5	3.0

* n/c—not calculated.

## Abbreviations

The following abbreviations are used in this manuscript:

CS: chitosan
PS: poly(styrene)
PAN: poly(acrylonitrile)
AN: acrylonitrile
S: styrene
IL: ionic liquid
FTIR: Fourier-transform infrared spectroscopy
AFM: atomic force micrsocopy
TGA: thermal gravimetric analysis
DSC: differential scanning calorimetry
TMC: trimesoyl chloride
PAMMAM: poly(amidoamine)
[bmim][BF_4_]: 1-butyl-3-methylimidazolium tetrafluoroborate
[bmim][PF6]: 1-butyl-3-methylimidazolium hexafluorophosphate
[bmim][Tf_2_N]: 1-butyl-3-methylimidazolium bis(trifluoromethylsulfonyl)imide
